# On the role of peptide hydrolysis for fibrillation kinetics and amyloid fibril morphology†

**DOI:** 10.1039/c7ra10981d

**Published:** 2018-02-13

**Authors:** Xinchen Ye, Mikael S. Hedenqvist, Maud Langton, Christofer Lendel

**Affiliations:** Dept. of Fibre and Polymer Technology, KTH Royal Institute of Technology Stockholm Sweden; Dept. of Molecular Sciences, Swedish University of Agricultural Sciences Uppsala Sweden; Dept. of Chemistry, KTH Royal Institute of Technology Stockholm Sweden lendel@kth.se

## Abstract

Self-assembly of proteins into amyloid-like nanofibrils is not only a key event in several diseases, but such fibrils are also associated with intriguing biological function and constitute promising components for new biobased materials. The bovine whey protein β-lactoglobulin has emerged as an important model protein for the development of such materials. We here report that peptide hydrolysis is the rate-determining step for fibrillation of β-lactoglobulin in whey protein isolate. We also explore the observation that β-lactoglobulin nanofibrils of distinct morphologies are obtained by simply changing the initial protein concentration. We find that the morphological switch is related to different nucleation mechanisms and that the two classes of nanofibrils are associated with variations of the peptide building blocks. Based on the results, we propose that the balance between protein concentration and the hydrolysis rate determines the structure of the formed nanofibrils.

## Abstract 2

Peptide hydrolysis determines the fibrillation rate and the morphology of amyloid-like nanofibrils formed by β-lactoglobulin at low pH.

## Graphical abstract

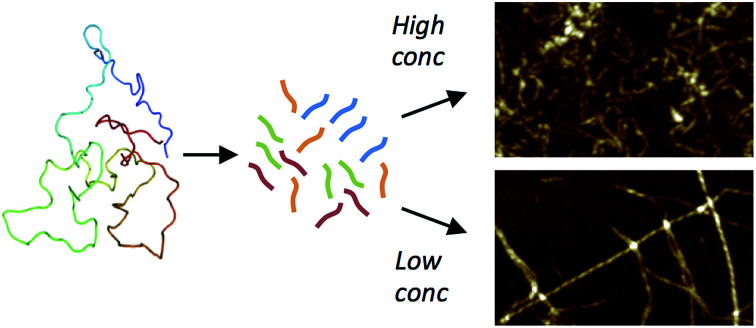

## Introduction

The self-assembly of proteins into highly ordered, β-sheet rich nanofibrils, so-called amyloid fibrils, has developed into a major area of research during the last decades. The main reason for this is the connection between these structures and a number of devastating diseases, including amyloidosis, Alzheimer's and Parkinson's diseases.^1^ However, an increasing number of examples where the amyloid-like structures are utilized in functional roles by living organisms show that protein nanofibrils (PNFs) *per se* are not pathological.^2,3^ This has opened the door of possibilities for what this class of structures can offer in nanotechnology and for the design of new biobased functional materials.^2,3^ The potential for this class of material is further strengthened by the fact that proteins from abundant natural raw materials, such as whey, can form PNFs.

The protein β-lactoglobulin from bovine whey has emerged as a useful and frequently studied model system, both for the fundamental mechanisms of nanofibril formation and for various materials applications.^2,4,5^ Formation of PNFs from β-lactoglobulin has been demonstrated in several studies, both by the purified protein and from whey protein isolate (WPI). β-Lactoglobulin forms amyloid-like fibrils under several different conditions, including high concentrations of urea,^6^ in the presence of alcohols,^7^ and at low pH and high temperature.^8,9^ Formation of PNFs at low pH and elevated temperature seem to be generic for many proteins of different sources, including *e.g.* soy protein,^10,11^ green pea protein,^12^ kidney bean phaseolin,^13^ cottonseed congossypin,^14^ and bovine caseins.^15^

WPI is an industrial scale raw material, which allows for large-scale applications. It is a mixture of proteins with β-lactoglobulin as the main component (typically between 50 and 60%).^16^ Fibrillation of WPI at low pH and high temperature has been suggested to proceed through hydrolysis of the proteins into smaller peptide fragments that spontaneously assemble into PNFs.^17–19^ Although other whey proteins, *e.g.* α-lactalbumin, can also form amyloid-like fibrils,^20^ PNFs formed under the applied conditions have been shown to be built exclusively from β-lactoglobulin-derived peptides.^21^ The fibril assembly has been correlated with the polypeptide hydrolysis reaction^18,22^ and mass spectrometry (MS) has been used to identify the peptide building blocks of the fibrils.^17,19,23^ The consensus of these investigations is that the two polypeptide segments, one in the most N-terminal part of β-lactoglobulin (residues 1–53) and one in the C-terminus (residues 138–162), constitute the key building blocks. The details of the fibril structure are, however, not known.

Furthermore, pure β-lactoglobulin has been reported to assemble into nanofibrils with distinct morphologies depending only on the initial protein concentration.^24^ In that study, low protein concentration (3%), resulted in long and straight fibrils while short, worm-like fibrils were formed at higher concentrations (7.5%). The authors showed that the morphological differences are correlated with differences in protein secondary structure but did not provide deeper insights in molecular determinants of the differences. We recently demonstrated that the same morphological switch is also observed for PNFs from WPI.^25^ Interestingly, we also found that the two classes of PNFs display different behavior in the assembly of micrometer-sized filaments. The fact that amyloidogenic polypeptides can form fibrils with different morphologies (strains) and also propagate the structural features through seeding is recognized.^26^ However, the very sharp morphological switch observed for β-lactoglobulin by only changing the initial protein concentration is intriguing.

In the present study, we provide further support for the role of hydrolysis as the rate-determining step in the fibrillation of β-lactoglobulin with WPI as starting material. We also dissect the chemical background of the morphological differences observed and propose that the origin of the concentration-dependent morphological switch is the hydrolysis process. These findings can facilitate the design of specific nanostructures for materials applications and provide new insights about the formation of disease-related amyloid *in vivo*.

## Experimental

### Sample preparations

WPI (Lacprodan DI-9224) with a total protein content of at least 92% was obtained from Arla Food Ingredients. According to the product specification, lactose and fat content are both below 0.2% and the rest of the dry weight is mainly salts. Samples for fibrillation were prepared by dissolving WPI in HCl, pH 2, to a final concentration of *ca.* 100 g l^−1^. The sample was then dialyzed against 10 mM HCl overnight using 6–8 kDa cut-off membrane (Spectrum Laboratories Inc.). Samples of different concentrations were prepared by dilution in 10 mM HCl with the assumption that no protein was lost during dialysis. No macroscopic aggregates were observed in these solutions. Fibrillation was carried out at 90 °C under quiescent conditions. For parts of the biophysical characterization described below the fibrils were purified through extensive dialysis using 100 kDa cut off membrane (Spectrum Laboratories Inc.) to separate the fibrils from low molecular weight peptides. The concentrations of protein samples for biophysical characterization in solution were adjusted to similar total protein concentrations based on absorbance at 280 nm and dry weight measurements. Samples for fibrillation at different initial pH values were prepared in the same way as described above but with initial dialysis against 3 mM HCl. The pH of each sample was then adjusted using 3 M HCl. Fibril seeds for the cross-seeding experiments were prepared either by 2 min sonication or by three freeze–thaw cycles of the fibril samples.

### SDS-PAGE

SDS-PAGE was carried out using precast Mini-Protean 4–20% gradient gels (Bio-Rad). Samples were mixed with Laemmli buffer (Bio-Rad) supplemented with 5 mM dithiothreitol and incubated at 90 °C for 10 min before analysis. The gels were stained by AcquaStain (Lubio science).

### Conductivity measurements

Conductivity of WPI samples and NaCl/CaCl_2_ reference samples was measured using a CDM210 instrument (MeterLab). WPI samples were prepared as described above, including dialysis. All samples contained 10 mM HCl in addition to the WPI or salt.

### Thioflavin T fluorescence

Samples for thioflavin T (ThT) fluorescence measurements were prepared by mixing 0.2 ml WPI solution with 2.4 ml 50 μM ThT solution. Fluorescence was measured at a Cary Eclipse Spectrofluorometer (Varian) with excitation at 440 nm and emission spectra recorded between 460 and 600 nm. The experiments were carried out with 2–3 replicates for each sample condition. Fibrillation kinetics obtained from ThT experiments were fitted to the Finke–Watzky 2-step model^27^ using Matlab software (see ESI for details†).

### Quantification of peptide formation

500 μl of each investigated sample was placed in a spin-filter with 10 kDa cut off (GE Healthcare). After spinning at 12 000 × *g* for 30 min the relative peptide concentration of the filtrate was measured using absorbance in the UV region. Measurements were repeated three times and three different wavelengths in the 220–240 nm region of the spectra were included in the final analysis.

### Atomic force microscopy

Samples for atomic force microscopy (AFM) were prepared by dilution (between 1 : 200 and 1 : 20 000) of the WPI samples in 10 mM HCl and then applied on freshly cleaved mica surfaces. After drying in air, the samples were investigated using a Dimension FastScan AFM (Bruker) operating in tapping mode. FastScan A cantilevers (Bruker) were used for the experiments and the images were investigated using Nanoscope 1.5 software. Control experiments were also performed in liquid using ScanAsyst liquid + cantilevers (Bruker) operating in peak force mode. These experiments showed the same difference between the morphologies of straight and curved PNFs.

### Fourier transform infrared spectroscopy

Samples were dried into films at 40 °C and then investigated using a Perkin-Elmer Spotlight 400 Fourier transform infrared (FTIR) imaging system equipped with a germanium attenuated total reflection (ATR) crystal. IR absorption spectra were recorded in ATR image mode for the region between 750 cm^−1^ and 4000 cm^−1^ with 16 scans and a resolution of 4 cm^−1^. FTIR spectra were deconvoluted into 9 Gaussian peaks in the amide I band (1580 cm^−1^ to 1700 cm^−1^) using fixed peak positions, an enhancement factor (*γ*) of 2 and a smoothing filter of 70%. The procedure and peak positions/assignments were the same as those used by Cho *et al.*^28^

### Circular dichroism spectroscopy

Samples were diluted in 10 mM HCl and circular dichroism (CD) measurements were carried out using a Chirascan instrument (Applied Photophysics) equipped with a Peltier temperature control system. The measurements were performed at 25 °C using a cell with 1 mm optical path length. The spectral region was recorded from 190 to 260 nm with a step size of 0.5 nm, a measurement time of 0.5 s per point and a bandwidth of 1 nm. The displayed spectra are averages of 5 individual scans for which the background signal has been subtracted and the amplitude corrected for the relative protein concentrations.

### Congo red absorbance spectroscopy

Samples were diluted 1 : 152 in 16 μM Congo red in phosphate buffered saline (PBS). The absorbance was measured between 400 and 700 nm in a plastic cuvette with 1 cm path length using a Cary 300 spectrophotometer (Varian).

### Protein auto-fluorescence spectroscopy

Samples (not diluted) were analyzed in a 3 × 3 mm quartz cuvette. Fluorescence emission spectra were recorded between 400 and 600 nm with excitation at 375 nm on a Cary Eclipse Spectrofluorometer (Varian).

### Mass spectrometry

Purified fibril samples were flash frozen in liquid nitrogen and lyophilized. Before MS analysis, the samples were dissolved in 8 M guanidinium hydrochloride with 0.1 M tris buffer pH 8 and 0.1 M dithiothreitol. 20 μl of this solution was added to 1 ml of 1 : 1 water/acetonitrile with 0.3% trifluoroacetic acid. Dimethoxy-4-hydroxycinnamic acid (SA) at a concentration of 10 g l^−1^ was used as matrix. The protein samples were mixed with the matrix solution in ratios between 1 : 10 and 1 : 1 (protein solution : matrix solution) and 1 μl was applied on a MPT 384 steel plate (Bruker). Matrix-assisted laser desorption/ionization time of flight (MALDI-TOF) spectra were acquired on an UltrafleXtreme instrument (Bruker) in positive reflection mode using a method optimized for the mass range 700–3500 Da. A peptide calibration standard from Bruker was used for calibration and the spectra were analyzed using FlexAnalysis 3.4 software. The peaks were assigned by comparison with previously published data.^17^

## Results and discussion

### Hydrolysis is the rate limiting process for WPI fibrillation

**Fig. 1:**
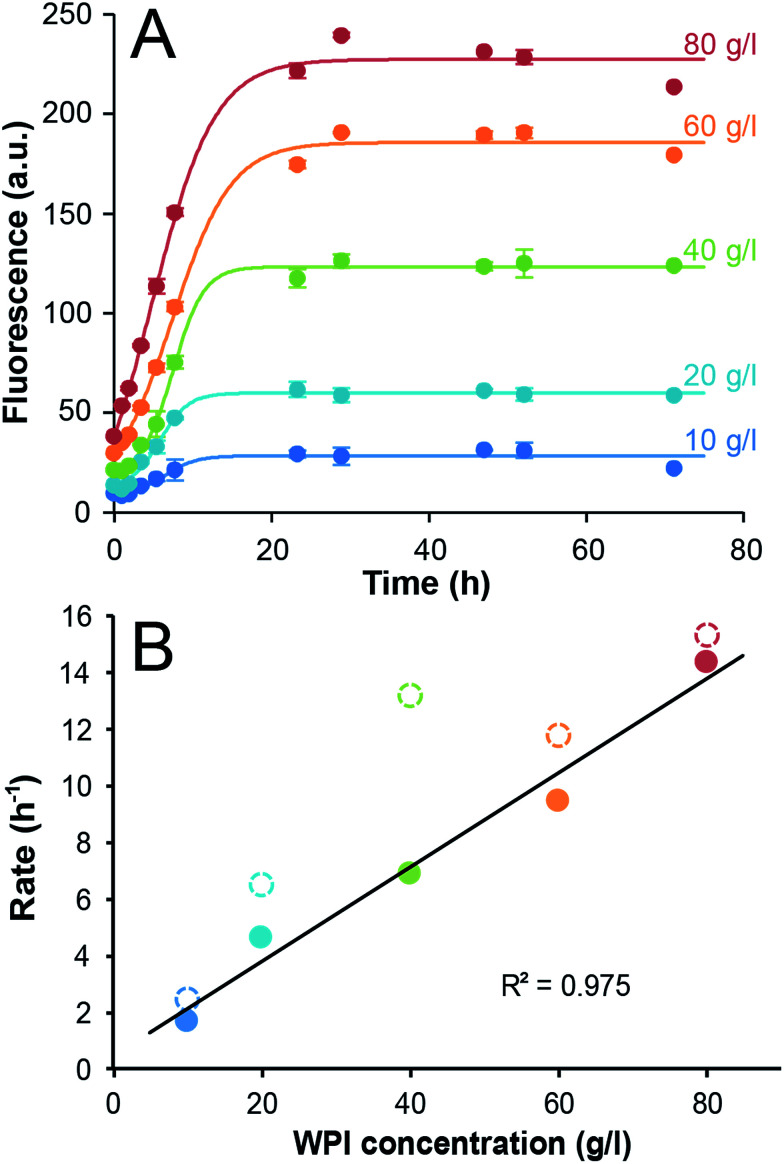
Fibrillation kinetics of WPI. (A) Time dependence of the ThT fluorescence at 485 nm for WPI solutions of 10–80 g l^−1^ incubated at 90 °C. The circles show the measured data (average of three samples) and the lines represents the best fits to the Finke–Watzky model. Error bars are ±1 standard deviation. (B) Initial rates, taken as the slope of the linear fits (ESI Fig. S4†) as function of initial sample concentration (filled circles). The max rates derived from the Finke–Watzky fit are shown for comparison (open circles). The colors of the data points correspond to those in (A). The black line shows the linear fit of the initial rates (*i.e.* only the filled circles).

Fibril formation in WPI solutions with initial concentrations ranging from 10 g l^−1^ to 80 g l^−1^ was monitored by ThT fluorescence. Conductivity measurements confirmed that changes in the protein concentrations did not significantly alter the salt concentrations (see ESI and Fig. S1†). The kinetic traces display a close to linear time dependence during the initial phase and then flatten out at their maximum values (Fig. 1A and ESI Fig. S2†). The absence of a distinct lag phase can be explained by the high temperature and high protein concentrations and similar features have been reported by others.^18^ Fitting the data using the Finke–Watzky 2-step model^27,29^ allowed us to estimate values of the lag time (*t*_lag_), the aggregation half-time (*t*_1/2_) and the maximum rate (see the ESI Table S1†). The fitted parameters confirm small values of *t*_lag_ (between 0.5 and 2.8 h) and reveal that *t*_1/2_ is between 5.9 and 8.3 h and without uniform dependence on protein concentration. The relationship between *t*_1/2_ and the initial monomer concentration provides information about the microscopic mechanisms of the fibrillation reaction.^30^ The fact that a double logarithmic plot of the measured *t*_1/2_*versus* initial WPI concentration (ESI Fig. S3†) does not follow any of the standard models for fibrillation suggests that the measured kinetics might reflect another process than the PNF assembly. To explore this further we applied a simplified approach where the initial reaction rates were estimated (by linear fits to the measured ThT signal during the first 8 h, ESI Fig. S4†) and we found a linear relationship between the initial rates and the WPI concentration (Fig. 1B). As a comparison, the maximum rates from the Finke–Watzky model are in the same range as the initial rates but slightly higher and with deviations from a linear correlation (Fig. 1B). Since the interpretation of the maximum rate for a system where the fibrillation is coupled to acid-catalyzed hydrolysis and a gradual pH change (*vide infra*) is not straightforward we chose to analyze the data by the simpler linear estimates of the initial reaction rates. Hence, while the change in ThT fluorescence appears to follow first-order kinetics with respect to the WPI concentration, the kinetic parameters *t*_lag_ and *t*_1/2_ are not significantly affected by the initial protein concentration. One possible explanation for this observation is that the self-assembly *per se* is not rate-limiting and that another step, for example some type of pre-processing is required for the protein to undergo the fibrillation process.

**Fig. 2:**
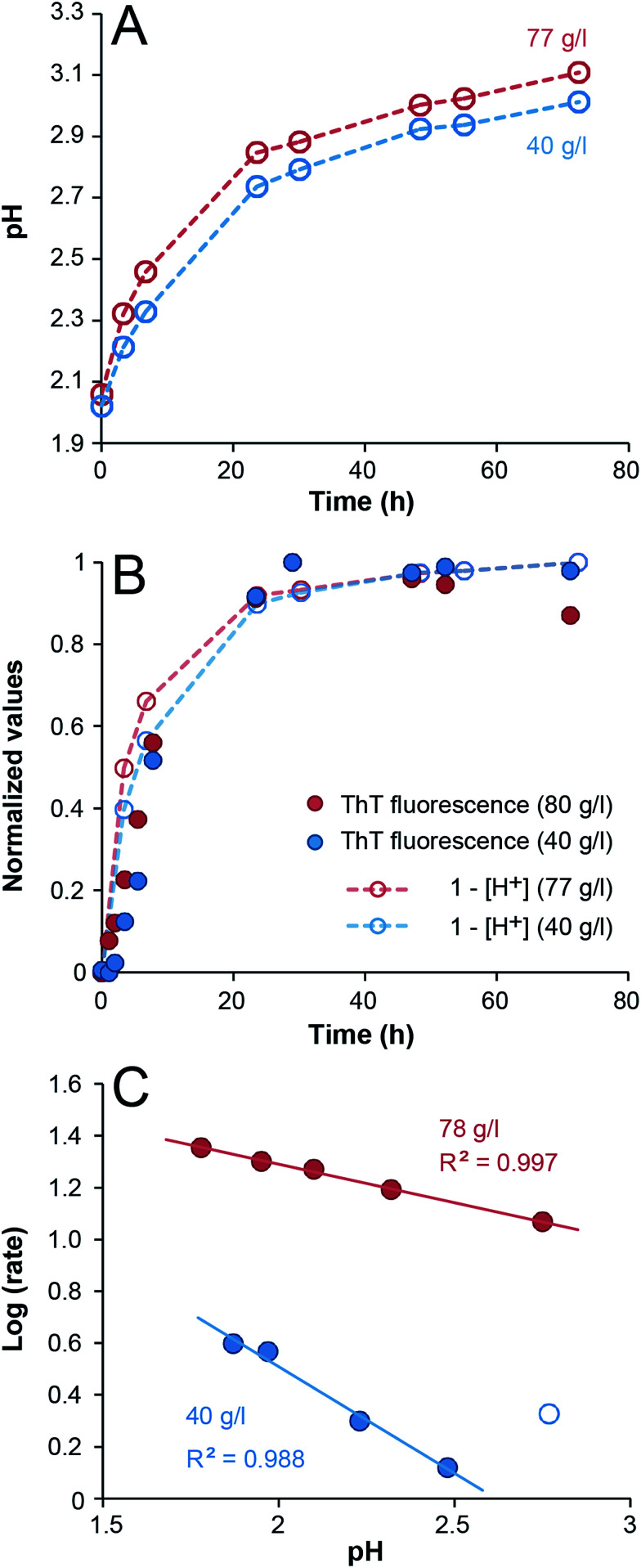
Changes in pH during WPI fibrillation and pH-dependence of the fibrillation kinetics. (A) Changes in pH of 40 g l^−1^ (blue) and 77 g l^−1^ (red) WPI samples during fibrillation. (B) The data from panel (A) displayed as normalized (1 − [H^+^]) together with normalized ThT fluorescence intensity (485 nm). (C) The logarithms of the initial fibrillation rates (in h^−1^) of 40 g l^−1^ (blue) and 78 g l^−1^ (red) WPI samples as function of pH. The 40 g l^−1^ sample at pH 2.8 (open blue circle) was not included in the linear fit (see the ESI† for details).

From previous studies it has been suggested that β-lactoglobulin undergoes peptide hydrolysis in order to assemble into nanofibrils.^17–19^ In particular, the peptide bonds of aspartate amino acids are sensitive to hydrolysis.^17,31^ SDS-PAGE of selected samples from the ThT experiments above confirms almost complete hydrolysis of the whey proteins into peptide fragments within the first 24 h of incubation (ESI Fig. S5†). Acid-catalyzed hydrolysis of peptide bonds is a reaction with first-order kinetics with respect to protein concentration and it is also expected to display first-order kinetics with respect to the H^+^ concentration. Notably, under the applied experimental conditions protein is in significant excess compared to H^+^ (the total amino acid concentration is around 0.1–1 M while [H^+^] = 0.01 M at pH 2). When monitoring the pH during the fibrillation reaction, a gradual increase was observed with time, starting at pH = 2 and gradually approaching *ca.* 3 (Fig. 2A). In fact, the time-scale of the H^+^ concentration changes is in good agreement with the ThT fluorescence curves (Fig. 2B). A perfect agreement is not expected because not every hydrolysis event leads to the formation of a peptide that can be incorporated in the fibrils. The fact that the initial slopes of the pH changes at high and low WPI concentrations are the same is in agreement with the large excess of protein compared to H^+^. No change in pH could be detected when samples without protein were incubated under the same conditions. We also investigated the pH-dependence of the initial fibrillation rate and there are indeed linear relationships between the initial pH of the WPI solutions and the logarithm of the fibrillation rate for both high and low WPI concentrations (Fig. 2C, ESI Fig. S6†). The decreased reaction rate with increased pH follows the same trend as reported for pure β-lactoglobulin^29^ and is in agreement with hydrolysis being the rate limiting step. However, changes in initial pH do not only affect the rate of hydrolysis. Altered electrostatic properties of the protein molecules and the solution can also affect the fibril morphology^29^ and the sol–gel transition. Indeed, the highest investigated pH (2.8) for the 40 g l^−1^ solution do not follow the linear correlation observed for the other samples (Fig. 2C, ESI Fig. S6†), indicating a change in the fibril assembly mechanism (see comment in the ESI†).

**Fig. 3:**
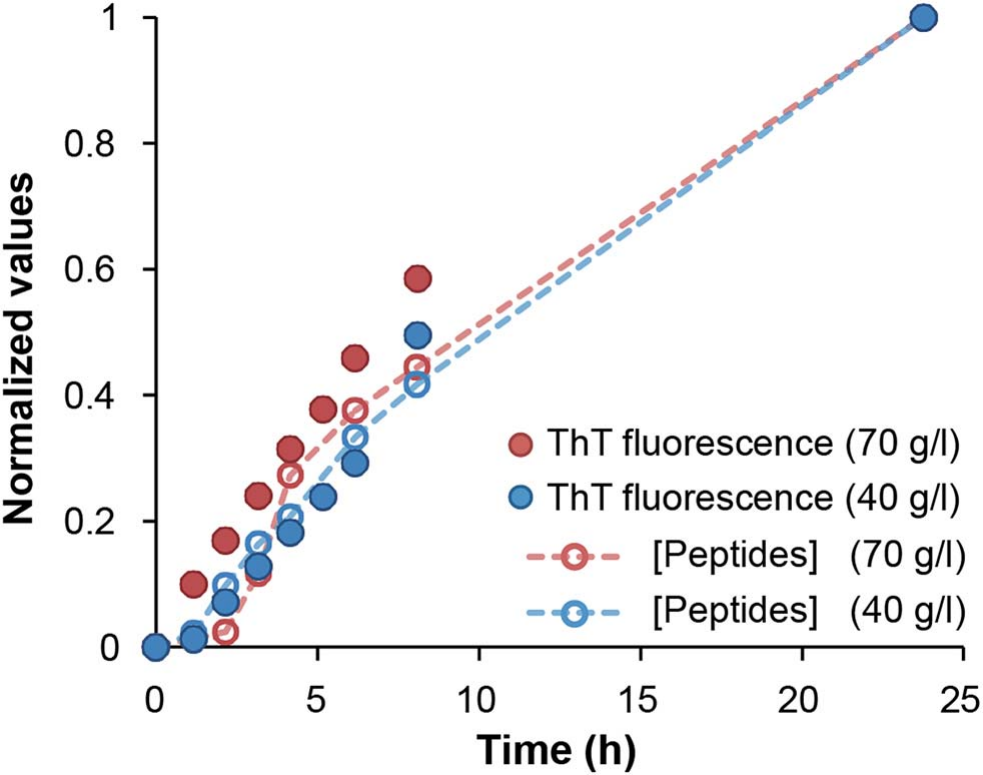
Comparison of normalized ThT fluorescence intensity and the formation of peptides <10 kDa in samples of 40 and 70 g l^−1^ initial WPI concentration, respectively.

Finally, we investigated the appearance of peptides <10 kDa in samples with 40 g l^−1^ and 70 g l^−1^ initial WPI concentrations. A comparison with the changes of ThT fluorescence in the same samples confirm that the hydrolysis and fibril formation processes occur on the same time scale (Fig. 3 and ESI Fig. S7†). Taken together, our data suggest that hydrolysis is the rate-determining step under the conditions investigated here, which is in agreement with previous studies of pure β-lactoglobulin.^17–19^

### A morphological switch for WPI fibrils as a function of starting concentration

**Fig. 4:**
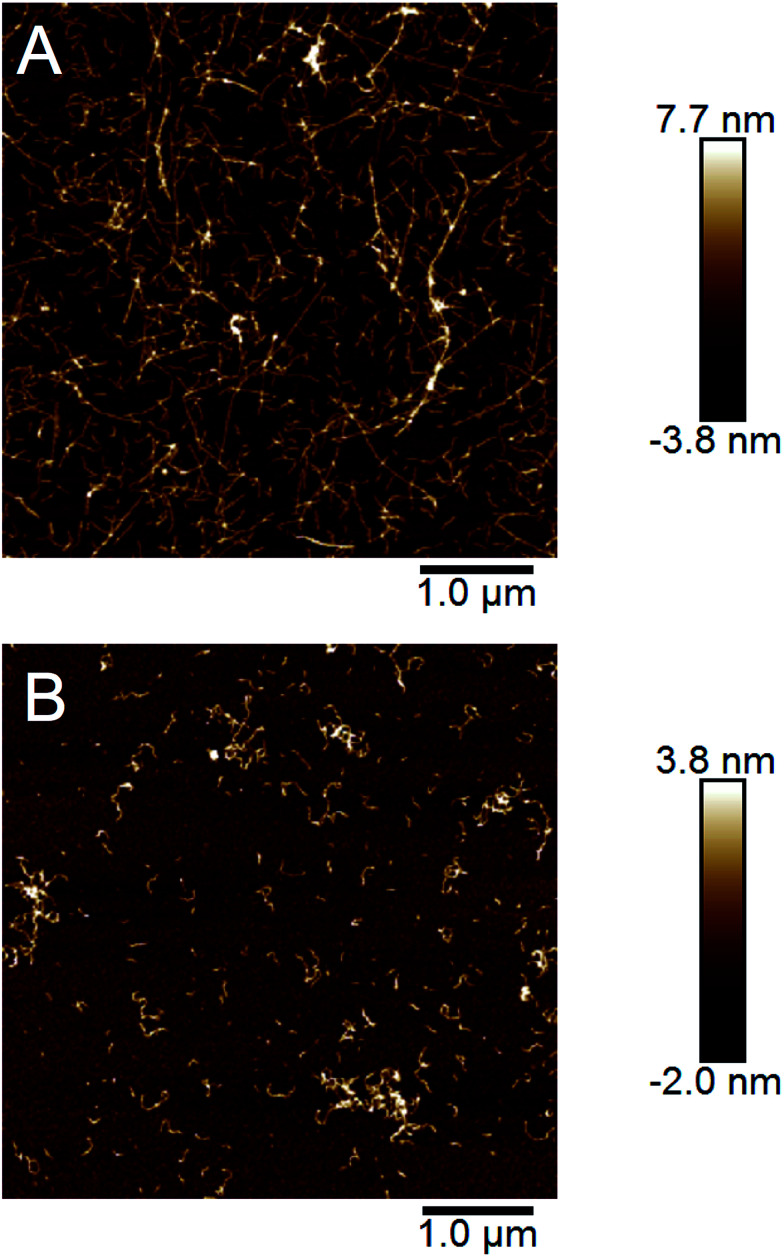
AFM images illustrating the morphologies of the fibrils formed at low and high initial WPI concentrations. (A) Straight and long fibrils formed in a 40 g l^−1^ WPI sample. (B) Curved and short fibrils formed in a 80 g l^−1^ WPI sample.

The end point samples from the fibrillation reaction were investigated using AFM. As previously reported by others for the fibrillation of pure β-lactoglobulin^24^ and recently also by us for WPI fibrillation,^25^ the morphologies of the fibrils formed at low and high concentrations differ. At lower concentrations (10–40 g l^−1^), straight fibrils that are up to a few micrometers long were observed (Fig. 4A, ESI Fig. S8†). However, in the two samples with the highest concentration (60 and 80 g l^−1^) the fibrils are shorter and more curved (Fig. 4B, ESI Fig. S8†). We have now observed this phenomenon for several sample series (ESI Fig. S9†) and for two separate batches of WPI raw material indicating that the phenomenon is highly reproducible.

In our previous work we analyzed a large set of AFM images in detail and found a significant difference in the persistence lengths for the two classes of PNFs.^25^ The persistence length of the straight fibrils was found to be *ca.* 1.9 μm while it is around 40 nm for the curved fibrils. These values are slightly lower than the ones reported for PNFs formed by pure β-lactoglobulin,^24^ but still the difference is of the same order of magnitude. Furthermore, the average heights of the fibrils were determined to be 4.1 ± 1.1 nm for the straight PNFs and 2.5 ± 0.5 nm for the curved PNFs.^25^ In summary, we find that the appearance of the straight fibrils we obtain is similar to the amyloid-like fibrils, also referred to as “semi-flexible fibrils”, previously reported for pure β-lactoglobulin by several groups (*e.g.*ref. 7, 21, 29, 32 and 33). The curved fibrils resemble those reported with altered solution conditions^7,8,29,34^ or at high initial concentration.^24^

### Both curved and straight fibrils have amyloid-like structures

**Fig. 5:**
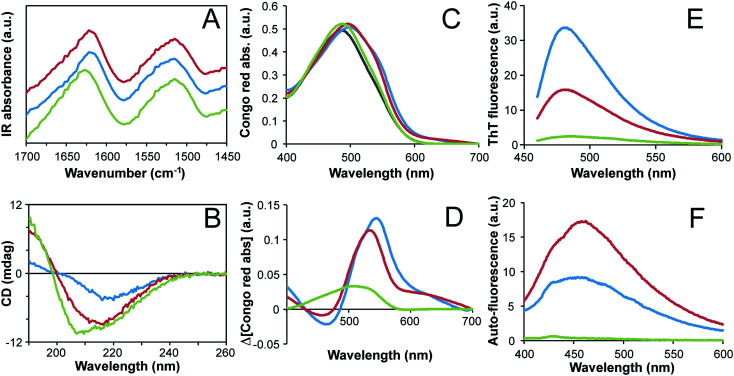
Structural characteristics of straight (blue lines) and curved (red lines) fibrils compared to non-fibrillar WPI at pH 2 (green lines). The fibrils were purified by dialysis and all protein solutions were adjusted to similar protein concentrations based on absorbance at 280 nm and dry weight measurements. (A) FTIR spectra measured on dry protein films. Data are scaled to show similar peak absorbances in the amide I region. (B) Far-UV CD spectra of protein solutions. (C) Congo red absorbance spectra. The grey line is Congo red in solution without any protein. (D) Changes in Congo red absorbance spectra compared to the dye in absence of protein. (E) ThT fluorescence spectra with excitation wavelength = 440 nm. (F) Protein auto-fluorescence spectra with excitation wavelength = 375 nm. All solution measurements were performed at pH 2, except for the Congo red absorbance that was performed in PBS buffer, pH 7.4.

The secondary structure contents of the fibrils formed at high and low concentrations were investigated using FTIR. The samples were first thoroughly dialyzed (100 kDa cut off membrane) to purify the fibrils and separate them from peptide fragments that are not incorporated in the fibrils. The samples were then dried into films before measurements. The FTIR spectra for the two classes of fibrils are similar (Fig. 5A). They show a shift towards smaller wavenumbers in the amide I region (*ca.* 1580–1700 cm^−1^) compared to non-fibrillar WPI, which is in agreement with an increased content of β-sheet structure and with previous FTIR studies of fibril formation from pure β-lactoglobulin.^7,8^ Further analysis of the amide I band by spectral decomposition^28^ confirms an increase in strongly hydrogen bonded β-sheet structure and a decrease in disordered and α-helical structure for the fibrils compared to the non-fibrillar WPI sample (ESI Fig. S10 and Table S2†). The results also suggest some differences between the two fibrillar morphologies, indicating a slightly higher β-sheet content and lower content of α-helices and disordered regions in the straight fibrils (ESI Fig. S10 and Table S2†). This is in agreement with the finding reported by vanderAkker *et al.*^24^ using vibrational sum-frequency generation spectroscopy.

To explore the secondary structure of the fibrils in solution, we measured the far-UV CD spectra of purified fibril samples. The minimum of the CD spectra for both types of fibrils shift from *ca.* 208 nm in the starting solution to *ca.* 215–220 nm after 3 days, which is in agreement with an increased content of β-sheet structure (Fig. 5B). The sample with straight fibrils displays a substantially lower amplitude than the curved fibrils sample, which can be attributed to scattering artifacts from the longer fibrils. Comparison of CD spectra for purified fibrils from shorter fibrillation times shows that the secondary structures of the two morphologies are similar but not identical (ESI Fig. S11†). At the shortest incubation time (3 h), the curved fibrils display a spectrum with the minimum point shifted towards longer wavelengths, while the situation is the opposite at longer fibrillation times (18 h and 72 h). Hence the secondary structure contents of the two fibril morphologies are similar but not identical.

To further investigate whether both types of fibrils are amyloid-like we studied the Congo red binding and the ThT fluorescence for the purified fibrils. These dyes preferentially bind to the repetitive β-sheet structure of amyloid fibrils and thereby experience alterations in their optical properties.^35^ Enhanced ThT fluorescence (Fig. 5E) as well as substantial shifts in the Congo red absorption spectrum (Fig. 5C and D) show that both morphologies are amyloid-like. The more intense ThT fluorescence and larger spectral shift of Congo red observed for the straight fibrils indicate a higher degree of structural order for that morphology, which is in agreement with the higher β-sheet content indicated by FTIR. Finally, protein auto-fluorescence associated with amyloid structures^36^ were also investigated. Again, the purified fibril samples display much higher fluorescence intensities than the non-fibrillar control (Fig. 5F). Interestingly, in this experiment the curved fibrils display higher emission intensity than the straight fibrils, which supports the conclusion that there are fundamental structural differences between the two morphological classes of PNFs.

Taken together, our AFM data confirm a morphological switch between initial WPI concentrations of 40 and 60 g l^−1^ and biophysical characterization of the fibrils shows that distinct structural features are associated with each of the morphologies although both can still be classified as amyloid-like. The difference in final length distributions for different starting concentrations could indeed be explained by changes of the relative importance of nucleation and elongation reactions.^37^ However, the morphological differences go beyond that and also involve molecular structure and mechanical properties.^25^

### Different peptide building blocks for the two fibril morphologies

**Fig. 6:**
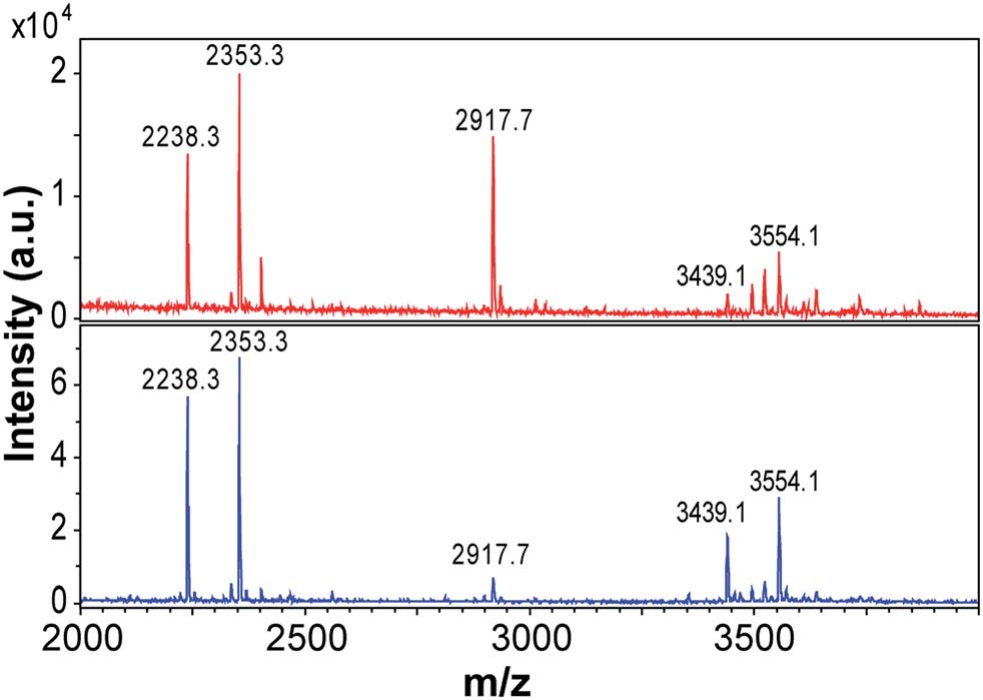
Peptide building blocks of PNFs identified by MALDI-TOF. The top spectrum (red) is for curved fibrils formed at 80 g l^−1^ and the bottom spectrum is for straight fibrils formed at 40 g l^−1^. The fibrils of the samples were purified and dissolved in 8 M GuHCl before analysis. The numbers are the molecular weights (in Da) of the corresponding peptide fragments carrying a single charge.

Previous studies using MS have shown that β-lactoglobulin fibrils are composed of peptide building blocks that primarily originate from the N-terminal and C-terminal segments of the protein.^17,19,23^ Similar regions have also been observed to play important roles to fibrillation of β-lactoglobulin at pH 7.0, 37 °C and 5 M urea.^38^ We investigated the composition of the purified WPI fibrils (72 h fibrillation time) using MALDI-TOF MS and the same protocol as employed by Akkermans *et al.*^17^ We found that the main peaks in the mass spectra correspond to the molecular masses reported for N-terminal segments 1–32/33 (3439/3554 Da) and 12–32/33 (2238/2353 Da) and for the C-terminal segment 138–162 (2917 Da) (Fig. 6). At low pH, hydrolysis preferentially occurs at aspartate residues in the polypeptide chain resulting in a number of distinct peptide fragments.^17,31^ The agreement between our results and the peptides reported for PNFs formed by pure β-lactoglobulin^17^ support the previous finding that fibrils formed from WPI are composed of β-lactoglobulin fragments.

Interestingly, the relative intensities of the MS peaks differ for the two morphological classes of PNFs. The straight fibrils seem to contain more of the N-terminal fragments than the curved fibrils (Fig. 6). After 3 days (72 h) of fibrillation the peak corresponding to the C-terminal fragment is very small for the straight fibrils while it is still of considerable intensity for the curved fibrils. Consistent differences were observed in four different sample preparations. These results suggest that the core building blocks of the two morphologies are different and suggest a higher degree of incorporation of the C-terminal fragment in the curved fibrils.

We repeated the MS experiments for samples with shorter fibrillation times (3 h and 18 h) (ESI Fig. S12†). After 3 h, the mass spectra of straight and curved fibrils are also rather similar in the detection region (2000–4000 Da). Potentially this could be related to the incorporation of longer variants of N-terminal fragments (*e.g.* residues 1–52/52, as reported by Akkermans *et al.*^17^). At longer fibrillation times, the mass spectra become more complex. This is in agreement with the longer hydrolysis time. After 18 h, obvious differences can be observed for the two fibril morphologies, in particular the relative intensities of the peak corresponding to the C-terminal segment 138–162 and those corresponding to peptides in the N-terminal region changes. Finally, after 72 h the peak corresponding to the C-terminal segment is very small for the straight fibrils while it is still one of the major peaks in the mass spectrum of the curved fibrils.

Although a more detailed MS investigation is required to explain all the details of the fibrillation process it stands clear that the two types of fibrils are build from different mixtures of the peptide components with curved morphology containing more of the C-terminal peptide. Similar results have previously been presented for fibrillation of ovalbumin at low pH.^39^ Their MS analysis of curved and straight fibrils revealed that the peptide compositions were different with a higher degree of incorporation of a slightly larger peptide fragment in the fibrils with curved morphology.

### Cross-seeding

**Fig. 7:**
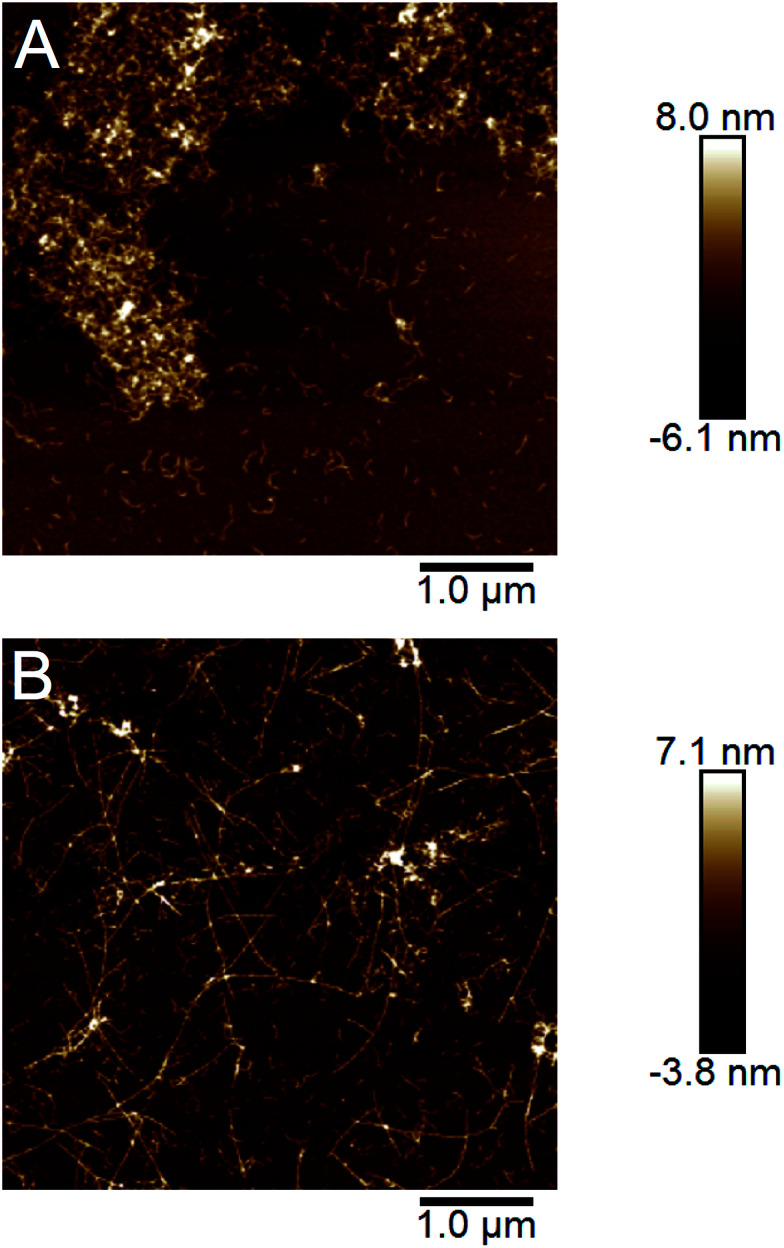
AFM images demonstrating the cross-seeding ability of the straight fibrils. (A) A sample with 77 g l^−1^ WPI fibrillated for 76 h. (B) The same WPI solution as in (A) with 5% seeds of straight fibrils added before fibrillation.

The ability to propagate specific structural features through seeding is one of the hallmarks of amyloid strains. We investigated the cross-seeding ability of the curved and straight fibrils, respectively. PNF samples with straight and curved fibrils, respectively, were prepared and used for seeding fresh solution of WPI by addition of 5% (relative protein content) of the fibrillated sample. The seed samples included both the PNF and residual non-fibrillar peptides and they were subjected to either 2 min sonication or three freeze–thaw cycles in order to break the PNFs into shorter fibril fragments. In all samples, the seeding resulted in changes in the sol–gel transitions. In particular the samples at high WPI concentration seeded with straight PNFs turned into a gel after *ca.* 2 h, which did not occur even after the longest fibrillation time (72 h) without seeds. Nevertheless, the gel-state samples could be re-dissolved and the fibril morphology investigated by AFM. The images reveal the presence of straight PNFs in the high concentration samples (Fig. 7, ESI Fig. S13†). In the low concentration samples, we observed a more frequent occurrence of curved fibrils but also some fibrils with straight morphology, indicating a competition between the two fibrillation pathways.

Loveday and co-workers previously investigated seeded WPI fibrillation using straight and curved fibrils prepared using different concentrations of CaCl_2_.^40^ They found that in a 20 g l^−1^ WPI solution incubated at pH 2 and 80 °C for 15 h, curved seeds seeded the formation of straight fibrils although some curved fibrils were also present. This is, at least in part, in agreement with our results and the slightly lower WPI concentration used by Loveday *et al.* might favor the formation of straight fibrils. No experiments with straight seeds added to WPI under conditions favoring the formation of curved fibrils were reported in that study.

### Molecular mechanism for the morphological switch

In this study we have shown that peptide hydrolysis is the rate-determining process for the formation of amyloid-like fibrils from WPI. This has previously been demonstrated for pure β-lactoglobulin^18^ and we confirm it to be the case also with WPI as starting material. Furthermore, we have observed that by simply changing the concentration of the starting material we can obtain fibrils with essentially different properties at all structure levels, from the molecular structure (Fig. 5) to the mechanical properties of macroscopic fibers.^25^ This morphological switch is intriguing and a better understanding of the associated mechanisms is of great value for the future design of protein-based nanomaterials as well as for the understanding of amyloid-related pathology.

We cannot completely exclude the possibility that the change in fibril morphology is related to crowding effects caused by the increased concentration of macromolecules. However, the presented MS data and biophysical characterization suggest that the explanation is rather to be found in the molecular events related to fibril nucleation. Moreover, the formation of straight fibrils in the cross-seeding experiments demonstrates that such structures can form also in the more crowded environment in samples with high initial WPI concentration.

The morphology of fibrils from β-lactoglobulin^41^ and other proteins, such as β2-microglobulin,^42^ have been shown to be dependent on solution pH and/or ionic strength. In previous studies, it has been reported that the formation of worm-like fibrils from β-lactoglobulin, similar to those we refer to as curved, occurs at low pH (<1.6)^29^ and in the presence of salt (NaCl, CaCl_2_)^8,29^ or alchohols.^7,34^ In these cases, the change in morphology can be explained by alterations in the intermolecular forces between the building blocks of the fibrils. In our experiments, the solutions were dialyzed before fibrillation and the salt concentration does not differ between high and low WPI concentrations (ESI Fig. S1†). The pH of the solution changes during fibrillation (Fig. 2A) but the difference between low and high concentration samples is small and unlikely to account for the sharp morphological switch. Hence, differences in ionic strength or pH are not likely to be the explanation for the morphological switch.

Our finding that peptide hydrolysis constitutes a key event in the fibrillation mechanism is in agreement with other studies. It has been shown for *e.g.* hen egg-white lysozyme,^43,44^ bovine casein,^15^ soybean proteins,^11^ and legume vicilins.^45^ Kroes-Nijboer *et al.*^18^ presented a theoretical framework for the fibrillation mechanism which proposed that the rate limiting process depends on a parameter *λ* = *k*_H_/(*k*_2_*C*_0_) where *k*_H_ is the rate of hydrolysis, *k*_2_ the rate of monomer addition to fibrils and *C*_0_ the total concentration of available building blocks (*i.e.* after full hydrolysis). The mechanistic breakpoint for when hydrolysis becomes rate-limiting is at *λ* = 1, which occurs between 5 and 10 g l^−1^ for pure β-lactoglobulin at 90 °C.^18^ With a 50–60% β-lactoglobulin content in WPI, this would correspond to 8–20 g l^−1^ WPI. Hence, it is not likely that the morphological switch occurring between 40 and 60 g l^−1^ is related to the switch in the rate-limiting mechanism of the fibrillation reaction.

Some clues about the mechanism for the morphological switch come from the study by Hamada *et al.*,^38^ which investigated the fibrillation of β-lactoglobulin at pH 7.0, 37 °C and 5 M urea. They found one N-terminal (residues 11–20) and one C-terminal (residues 127–142) region, corresponding to parts of the identified peptides in our MS experiments, which play important roles in the fibrillation process. Interestingly, when using fibril seeds from the C-terminal peptide, fibrils formed by a full-length variant of β-lactoglobulin adopted a “curly” morphology while seeds from the N-terminal segment, resulted in straight fibrils.^38^ Our MS analysis indicates that the curved fibrils from WPI have a higher relative amount of the C-terminal peptide (residues 138–162) than what is found in the straight fibrils. Hence, it seems that the degree of incorporation of this C-terminal segment in the fibrils could be a reason for the switch in fibril morphology.

**Fig. 8:**
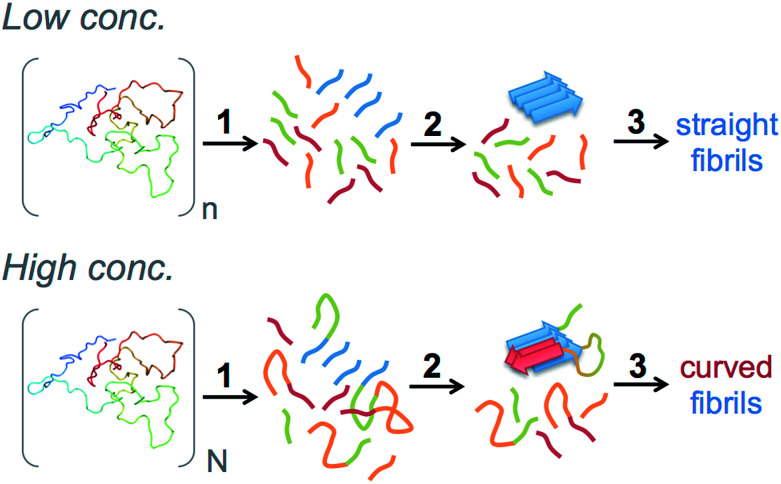
Schematic illustration of the proposed molecular mechanism for the morphology switch. The arrows indicate hydrolysis (1), nucleation (2) and fibril elongation (3). At low starting concentration, hydrolysis results in peptide fragments with a length distribution that, on average, is shorter than at high concentrations. This affects the structure of the fibril nuclei as longer peptide segments will be included and the C-terminal core segment will be incorporated to a higher degree at higher starting concentrations.

But why does increased initial WPI concentration lead to higher incorporation of the C-terminal segment? Here we want to make the connection back to the role of peptide bond hydrolysis in the fibrillation process. With an increasing protein concentration there will be a shift in average size distribution towards longer peptide fragments and also a higher concentration of polypeptides in which the N- and C-terminal amyloid core segments are still linked together. Such linkage may be realized either through an intact full-length protein or by the native disulfide bond between Cys66 and Cys160. We hypothesize that nucleation from longer peptide fragments leads to a different structural organization of the fibril nuclei and potentially higher incorporation of C-terminal segments, which then leads to a different morphology of the fibrils (Fig. 8). Hence, we propose that peptide hydrolysis is not only rate limiting in this system – it is also the explanation for the morphological switch.

Our hypothesis is supported by the study of Gao *et al.*,^46^ which showed that pretreatment of whey protein with different proteases resulted in alterations the morphology of the nanofibrils formed at pH 2 and 90 °C. Peptide hydrolysis has also been suggested to have a key role in the formation of multistranded amyloid ribbons from β-lactoglobulin and hen egg white lysozyme as the appearance of such structures correlated with the degradation of the full length proteins into small peptide fragments.^39^ That study does not, however, investigate the molecular organization of the protofilaments that assembles into the larger ribbon structures. Notably, no multistranded ribbons were observed in any of our samples. A morphological switch similar to the one we observe has been described for hen egg white lysozyme fibrils at pH = 2.^47^ The switch between “rigid fibrils” and “oligomers/curvelinear fibrils” (with some similarities of our straight- and curved fibrils, respectively) was described as a phase transition and modeled using colloidal charge repulsion. Our hypothesis provides a molecular perspective of the process but does not contradict a similar model for β-lactoglobulin. However, critical parameters such as the average charge and the stoichiometry of the aggregates, depends on the lengths of the peptide fragments, and thereby also on the initial protein concentration. Notably, peptide hydrolysis was not included in the model suggested for hen egg white lysozyme despite the fact that other studies have suggested it to be the origin of different aggregate morphologies.^44^

## Conclusions

In this study we have shown that peptide hydrolysis is the rate-determining step for the formation of β-lactoglobulin nanofibrils from WPI at pH 2 and 90 °C. We have also investigated the molecular mechanism behind the concentration-dependent switch of fibril morphology previously observed by us and others. The presented results show that the two classes of fibrils have distinct structural properties although both can be classified as amyloid-like. We propose a mechanism where the structure and morphology of the fibrils, at least under the investigated conditions, are determined by the extent of peptide hydrolysis. Changes in the composition of the peptides formed by hydrolysis alter the structure of the fibril seeds and result in nanofibrils with distinct morphologies. Hence, our findings highlight the role of peptide hydrolysis for the rate of the fibril formation process as well as the structure of the formed fibrils. These insights open for the possibility to control the functional properties of protein nanomaterials through the processing conditions, *e.g.* by employing proteases with different substrate specificities. Our conclusions may also be of relevance for amyloid pathology as they suggest that not only the total concentration of an amyloidogenic protein is an important parameter. The processing capacity in relation to the total concentration could in fact determine what type of aggregates that are formed.

## Conflicts of interest

There are no conflicts to declare.

## Supplementary Material

RA-008-C7RA10981D-s001
