# Supplementary material for: On the role of peptide hydrolysis for fibrillation kinetics and amyloid fibril morphology
Source: RSC Adv. 2018 Feb 13;8(13):6915–24. doi: 10.1039/c7ra10981d (PMC9078321; doi:10.1039/c7ra10981d)
Supplement: RA-008-C7RA10981D-s001 [file RA-008-C7RA10981D-s001.pdf]

Electronic Supplementary Material (ESI) for RSC Advances.
This journal is © The Royal Society of Chemistry 2018

Supplementary Information

On the role of peptide hydrolysis for fibrillation kinetics and amyloid fibril morphology

Xinchen Ye, Mikael S. Hedenqvist, Maud Langton, Christofer Lendel

Salt concentration is not affected by the protein concentration

The morphology of β-lactoglobulin fibrils can be altered by the ionic strength of the solution. [1, 2] To exclude that the morphology switch described in the present work originates from differences in salt concentrations between the samples with low and high protein concentration we measured the conductivity of samples before fibrillation. Reference curves were measured for various concentrations of NaCl and $CaCl_2$ in 10 mM HCl (blue and red circles in Fig. S1). Samples containing 10 – 73 g/l WPI in 10 mM HCl (after dialysis) were also measured and the conductivity was found to be between 3.2 and 3.6 mS/cm (indicated by green lines in Fig. S1). As comparison, the conductivity corresponding to 50 mM NaCl and 33 mM $CaCl_2$ (ca 9.5 mS/cm) is indicated with a black broken line in Fig. S1. According to Loveday *et al.* these salt concentrations represents the critical ionic strength for the formation of curved fibrils.[1] Clearly, the salt concentration of all samples used in our study is below that.

Fitting ThT fluorescence data to the Finke-Watzky 2-step model

The time-dependent ThT fluorescence data was fitted to the Finke-Watzky 2-step model.[3] This model was selected because it represent a "simplest possible" approach and the aim of the study was not to dissect the details of the fibrillation kinetics, as that has been thoroughly examined in other work (e.g. Ref. 4). Assuming that the fluorescence signal is proportional to the concentration of fibrils, *[B](t)* where *t* is time, the data was (after subtraction of the fluorescence intensity at $t = 0$) fitted to the following equation:

$$[B](t) = A_0 - \frac{\frac{k_1}{k_2} + A_0}{1 + \frac{k_1}{k_2 A_0} \exp\left[(k_1 + k_2 A_0)t\right]} \quad (1)$$

As the model does not incorporate any pre-processing step (hydrolysis) the original interpretations of the fitted parameters A_0, k_1 and k_2 do not apply here. Nevertheless, the model can be used to derive values for the "lag time" (t_{lag}), the time at which the signal has reached half of its maximum intensity ($t_{1/2}$) and the maximum rate of change (max slope of the fitted curve) using the following expressions:[1]

$$t_{lag} = \frac{1}{k_1 + A_0 k_2}\left(\ln\left(\frac{A_0 k_2}{k_1}\right) - 4\frac{A_0 k_2}{k_1 + A_0 k_2} + 2\right) \quad (2)$$

$$t_{1/2} = \frac{\ln\left(2 + \frac{A_0 k_2}{k_1}\right)}{k_1 + A_0 k_2} \quad (3)$$

$$max\,rate = \frac{\left(\frac{k_1}{k_2} + A_0\right)(k_1 + A_0 k_2)}{4} \quad (4)$$

Comment to Figure 2C and Supplementary information Figure S6

The fibrillation kinetics of the 40 g/l sample at pH 2.8 (data point shown as open blue circle in Figure 2 of the main text and as open red circles in Fig. S6A and S6C) displayed a different behavior than the other samples. All 40 g/l samples except the one at pH 2.8 have a common point of intersection (Fig. S6A) and the same thing is observed for the samples at 78 g/l (Fig. S6B). This indicates that the pH also affects other steps in the self-assembly process. For example, a deprotonation of a carboxyl group is not unlikely in this pH region and could allow alternative assembly pathways and thereby significantly change the apparent kinetics.

Table S1. Kinetic parameters for WPI fibrillation derived from the Finke-Watzky model.

WPI (g/l)	t_{lag} (h)	$t_{1/2}$ (h)	max rate (h^{-1})	Adjusted R^2
10	2.8	6.7	2.4	0.900
20	2.4	5.9	6.5	0.991
40	3.7	7.6	13.3	0.996
60	1.7	8.3	11.8	0.995
80	0.5	6.6	15.4	0.992

Table S2. Detailed results from the deconvolution of FTIR data.

Secondary structure		Relative area of Gaussian components (%)		
Position (cm^{-1})	Assignment	Curved	Straight	Non fibril
1618	β-sheets, strongly hydrogen-bonded peptide group	45.6	54.6	47.4
1625	β-sheets, strongly hydrogen-bonded peptide group	24.1	17.0	4.6
1634	β-sheets, weakly hydrogen-bonded peptide group	1.8	1.9	8.3
1644	Unordered	5.3	4.3	9.6
1651	α-helices and random coils	3.5	3.0	5.2
1658	α-helices	3.7	3.4	6.0
1667	β-turns	11.9	10.8	11.6
1680	β-sheets, weakly hydrogen-bonded peptide group	1.2	1.3	4.2
1691	β-turns	2.9	3.7	3.1

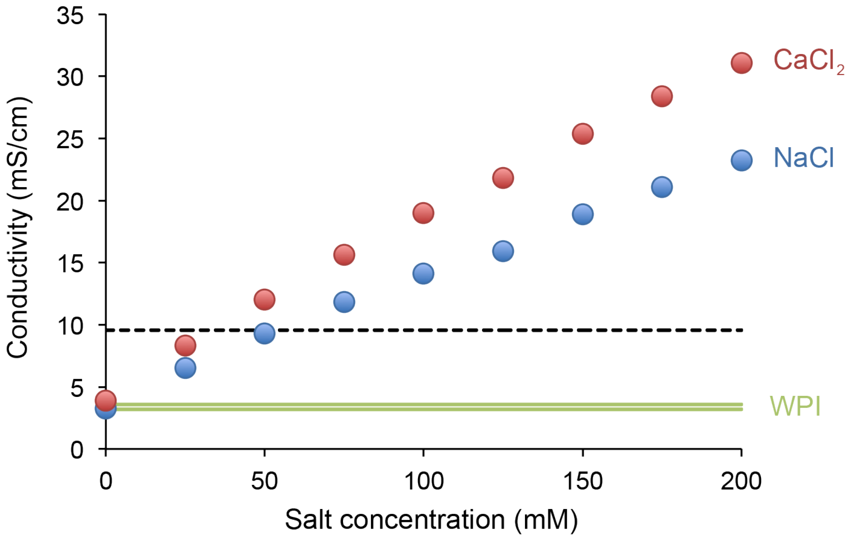

Figure S1. Conductivity measured for 10 mM HCl solutions with different concentrations of NaCl (blue circles) and $CaCl_2$ (red circles), respectively. The two green lines indicate the range of the conductivity measured in dialyzed WPI samples with 10 – 72 g/l concentrations in 10 mM HCl (no salt added). The black broken line correspond to the critical salt concentration for the formation of curved fibrils according to Loveday *et al.*[1]

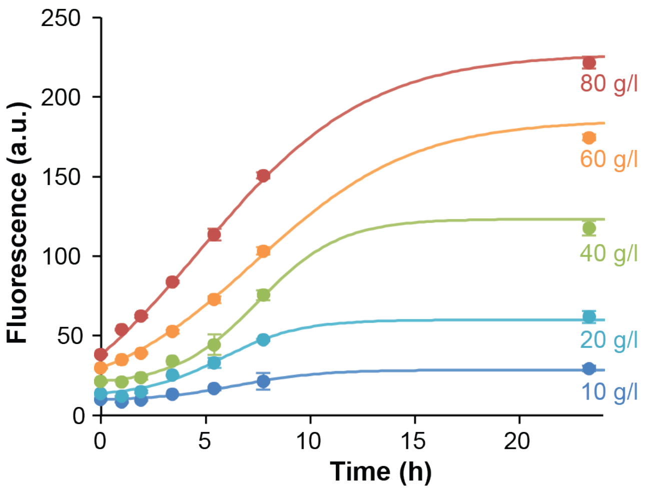

Figure S2. Fibrillation kinetics of WPI. Time dependence of the ThT fluorescence at 485 nm for WPI solutions of 10 – 80 g/l incubated at 90 °C. The circles show the measured data and the lines represent the best fits to the Finke-Watzky model. Error bars are ± 1 standard deviation. Same data as in shown in Fig. 1 in the main text but zoomed in on the first 24 h.

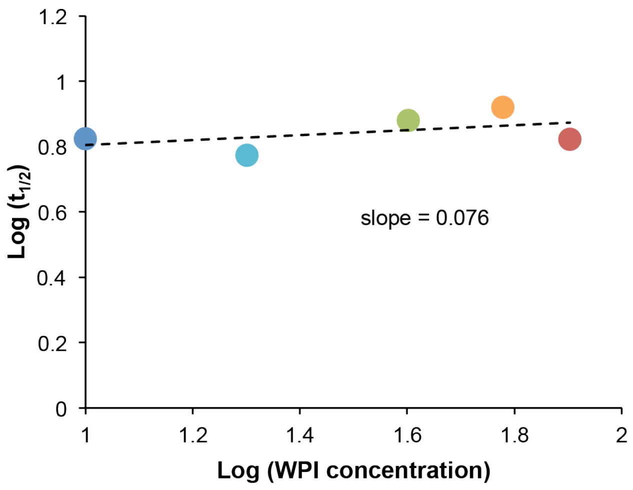

Figure S3. Double logarithmic plot of the $t_{1/2}$ values versus initial WPI concentration. The slope of the linear fit to the data is indicative of the underlying fibrillation mechanism and is typically in the range −0.5 or lower.[5] For our data we obtain a slope close to zero, or even slightly positive. The colors of the data points correspond to the curves in main text Fig. 1 and Fig. S2.

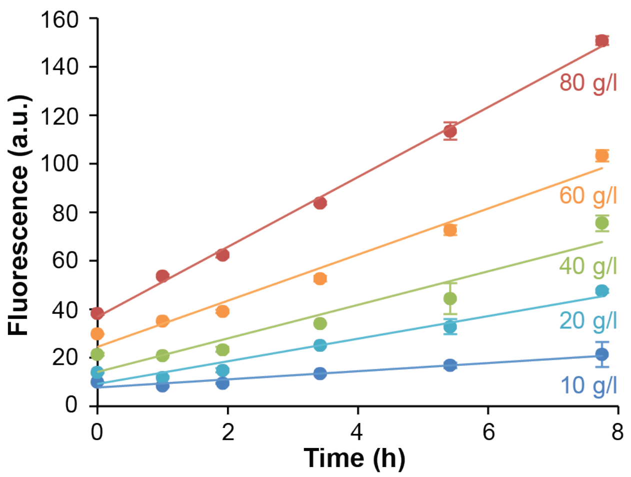

Figure S4. Fibrillation kinetics of WPI. Linear fits to the ThT fluorescence data of the first 8 h of fibrillation. Coefficients of determination, R^2, for the fits are between 0.910 and 0.998.

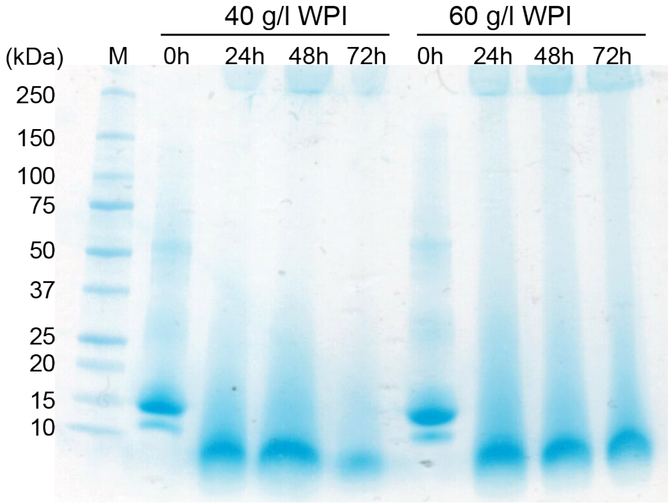

Figure S5. SDS-PAGE of selected samples from the fibrillation experiments shown in Fig. 1 in the main text and Fig. S2. Initially (0 h) the main protein components of WPI, β-lactoglobulin (18.4 kDa) and α-lactalbumin (14.1 kDa), are clearly visible and more weakly serum albumin (69 kDa). After incubation at 90 °C for 24 h or more, the samples are hydrolysed and mainly contains peptides shorter than 10 kDa. There are also signs of large aggregates that are unable to enter the gel in the incubated samples.

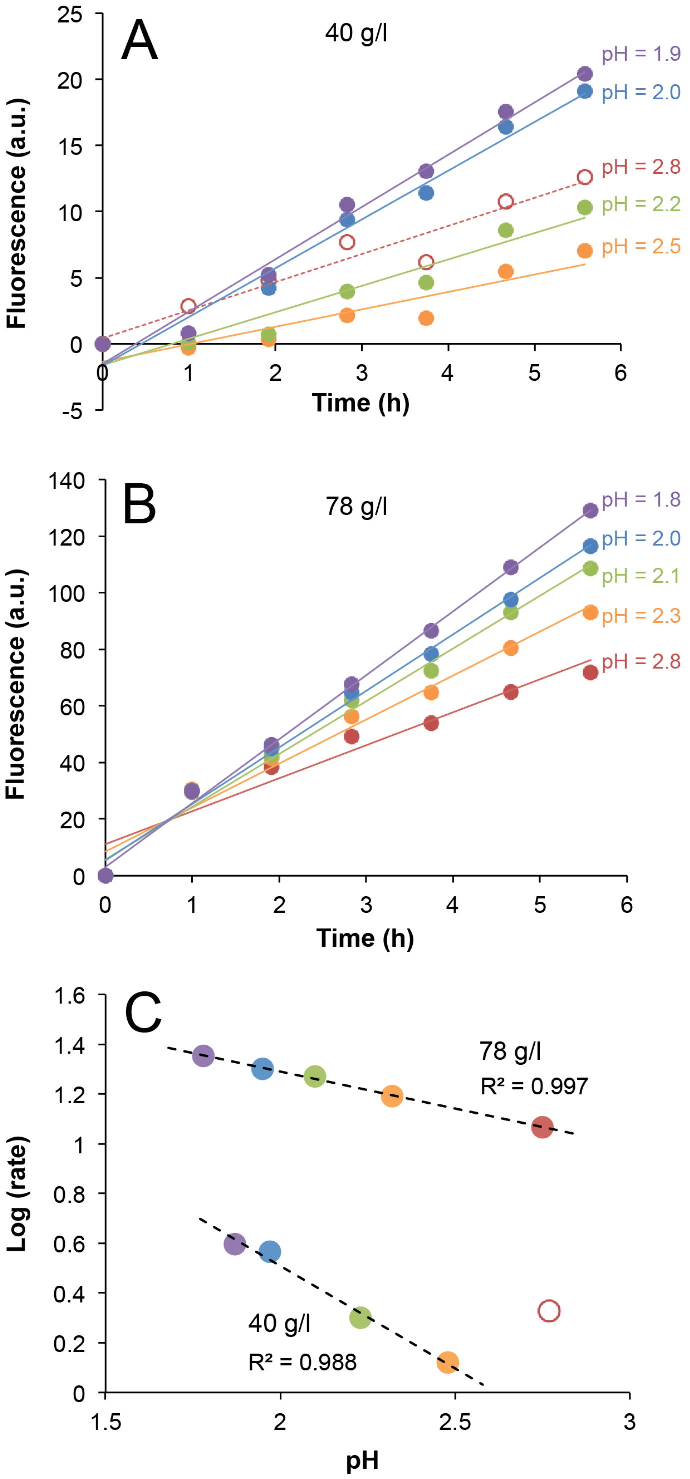

Figure S6. The pH-dependence of WPI fibrillation kinetics investigated by ThT fluorescence. **(A,B)** Linear fits to the kinetic data (ThT fluorescence at 485 nm) of the first 5.5 h of fibrillation of 40 g/l (A) and 78 g/l (B). The initial rates are taken as the slope of the fitted lines. R^2 values for the fits are between 0.920 and 0.998, except for the orange data set (pH 2.5) in panel (A) that has a $R^2 = 0.857$. **(C)** The logarithm of the rates (in h^{-1}) extracted from (A) and (B) plotted against initial pH of the samples. The 40 g/l sample at pH 2.8 displayed different kinetic behaviour and was not included in the linear fit (*vide supra*). The colors of the data points correspond to the curves in (A) and (B).

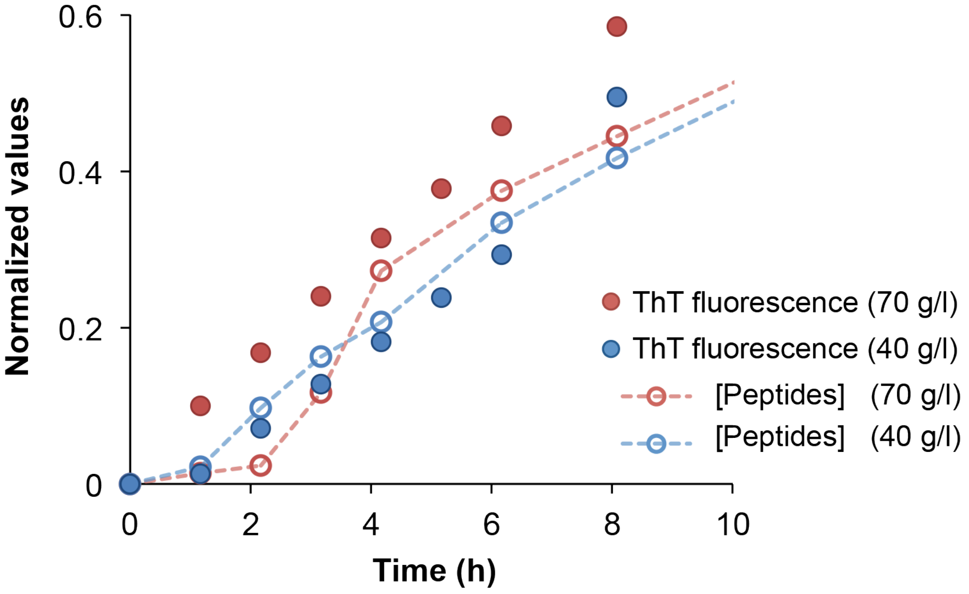

Figure S7. Comparison of normalized ThT fluorescence intensity and the formation of peptides < 10 kDa in samples of 40 and 70 g/l initial WPI concentration, respectively. The data is the same as in Fig. 3 of the main text. The correlation between the increase in ThT fluorescence and peptide formation is good for the 40 g/l sample while the peptide formation rate seems to be slightly slower than the development of ThT fluorescence for the 70 g/l sample. One explanation for this may be that hydrolysis initially leads to the formation of polypetides > 10 kDa that will not be detected in the employed spin-filter assay.

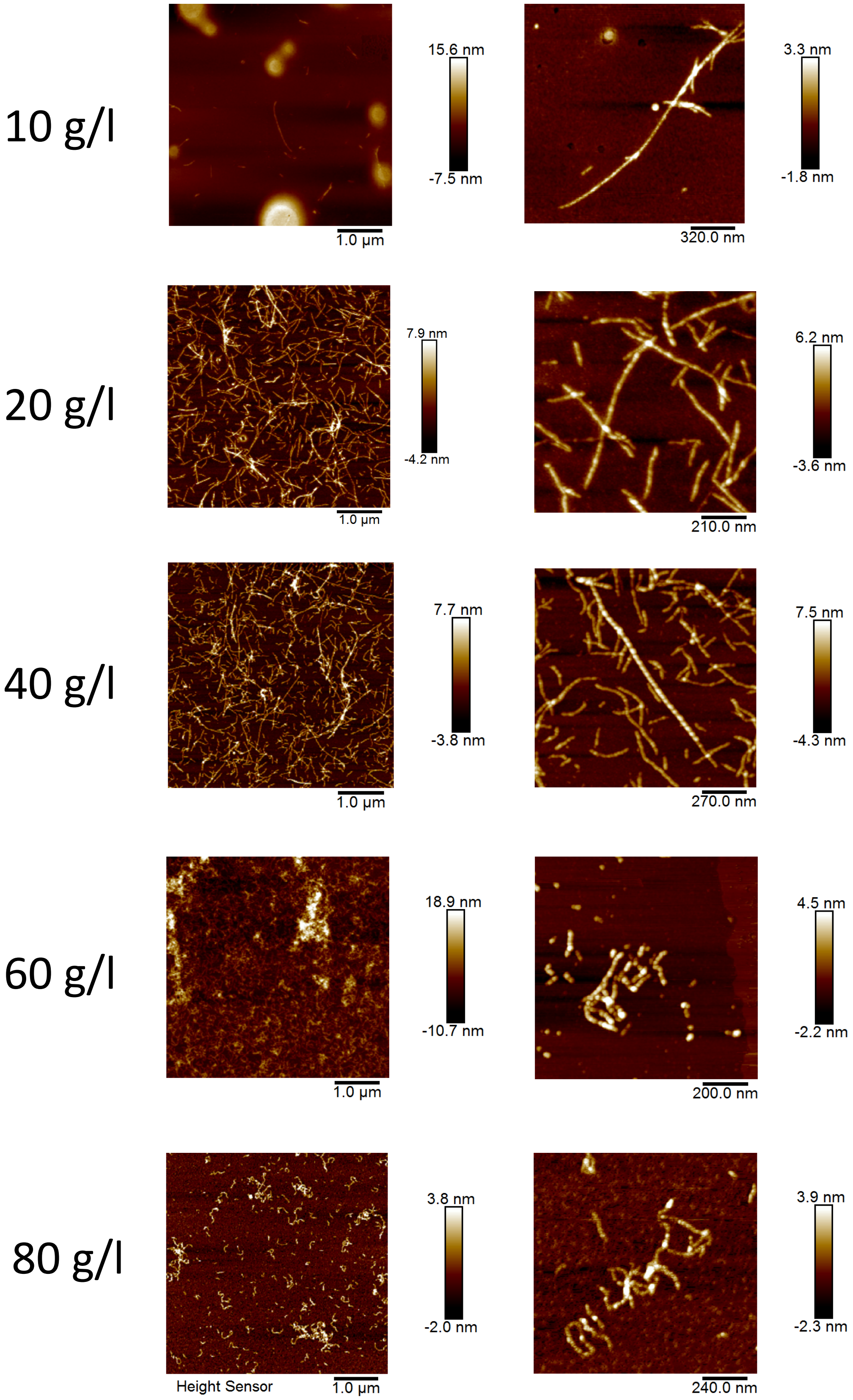

Figure S8. AFM images of fibrils formed in solutions of 10 – 80 g/l initial WPI concentration.

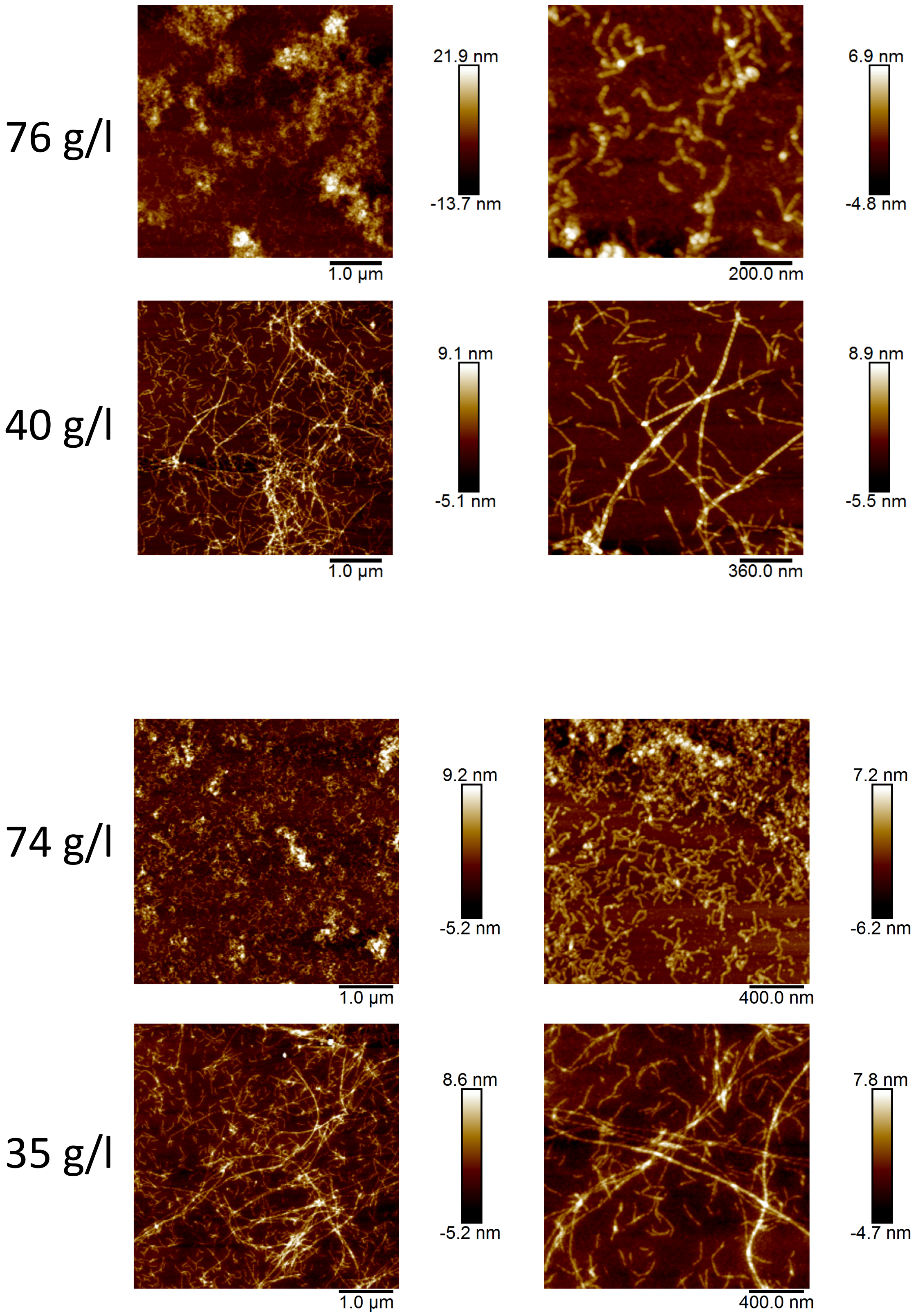

Figure S9. AFM images from two additional sample batches (upper four images from one batch and lower four images from another batch) confirm a morphology difference or PNFs formed at high and low initial WPI concentration.

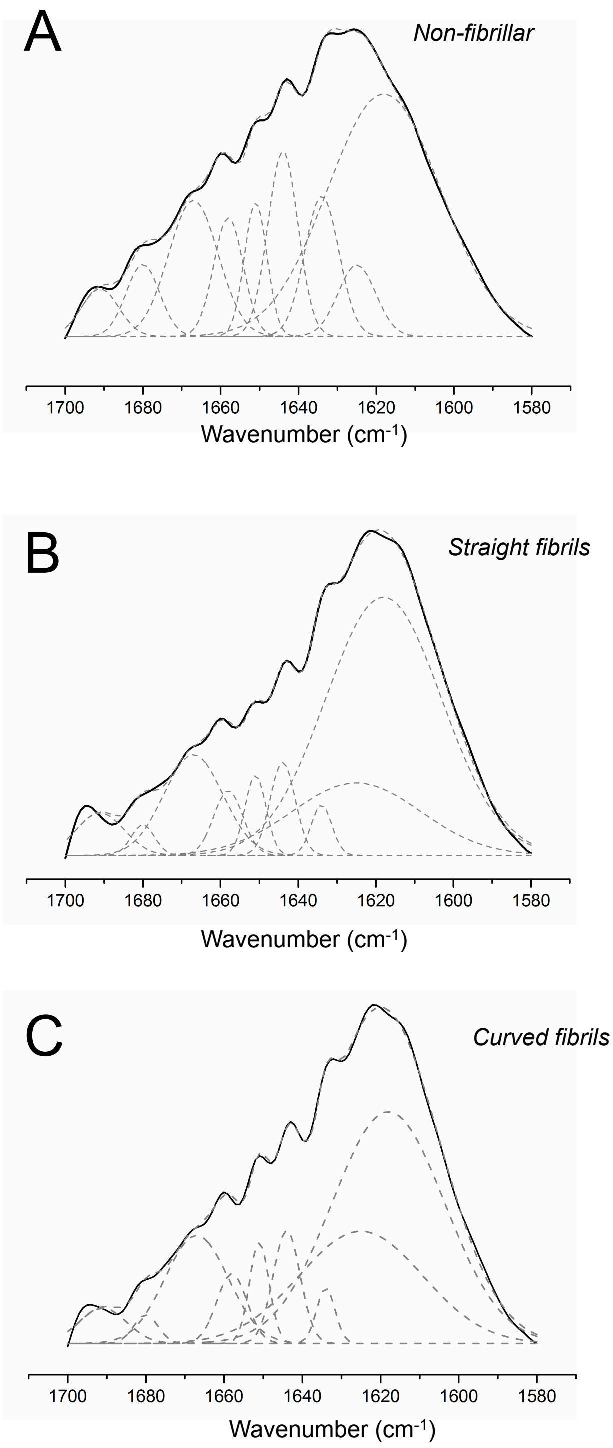

Figure S10. FTIR spectral deconvolution. The amide I band was deconvoluted into 9 gaussian components as previously described.[6] The original spectra and the best-fit deconvolutions are shown for a non-fibrillar control **(A)**, straight fibrils formed at low WPI concentration **(B)** and curved fibrils formed at high WPI concentration **(C)**. A detailed summary of the results is presented in Table S2.

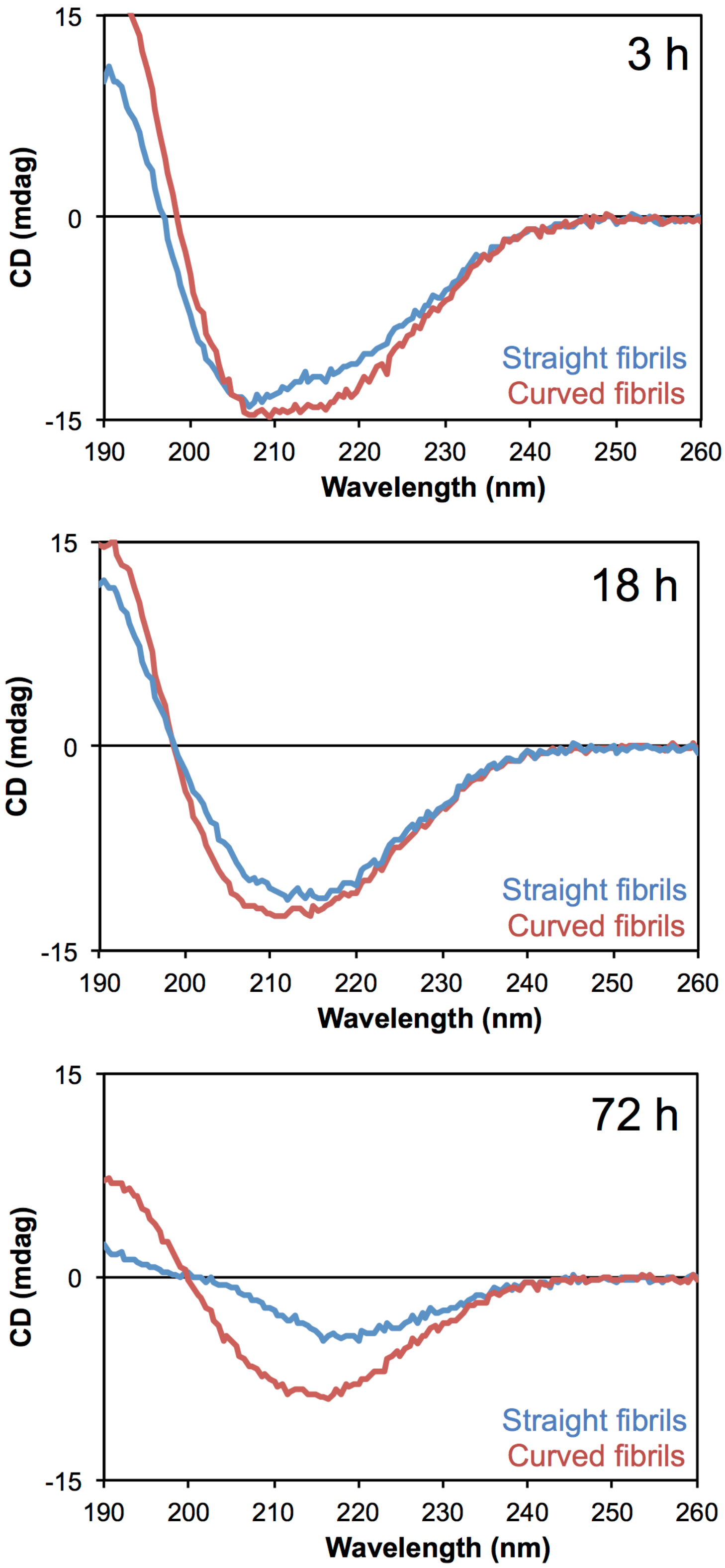

Figure S11. Far-UV CD spectra of purified fibrils/aggregates formed in 40 g/l (blue) and 80 g/l (red) WPI solution, respectively. Samples were incubated for 3 h, 18 h and 72 h (end of fibrillation) as indicated. The 72 h spectra are the same as shown in Fig. 5B in the main text.

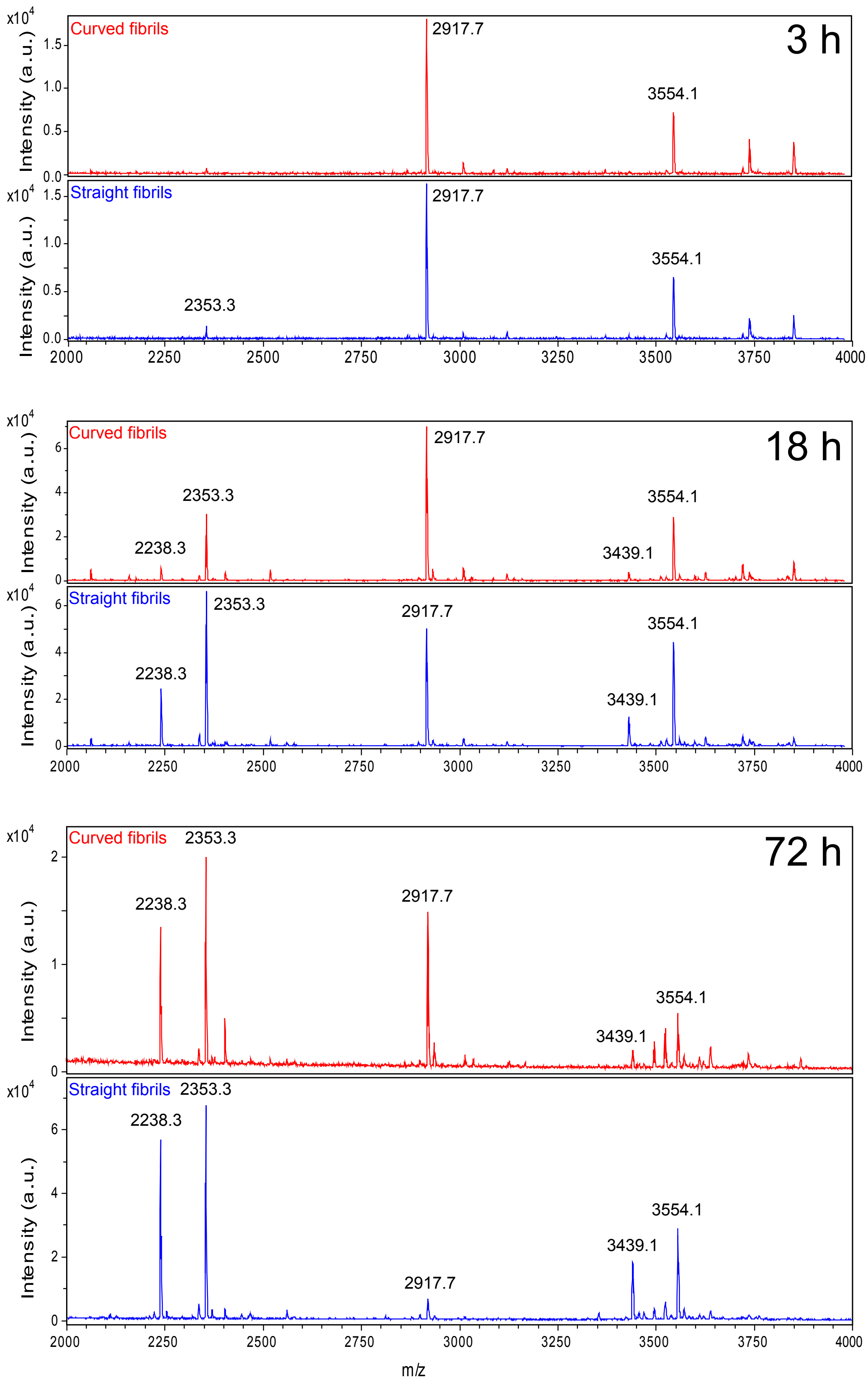

Figure S12. MALDI-TOF spectra of purified fibrils/aggregates formed in 40 g/l (blue) and 80 g/l (red) WPI solution, respectively. Samples were incubated for 3 h, 18 h and 72 h (end of fibrillation) as indicated. The 72 h spectra are the same as shown in Fig. 6 of the main text. The fibrils of the samples were purified and dissolved in 8 M GuHCl before analysis.

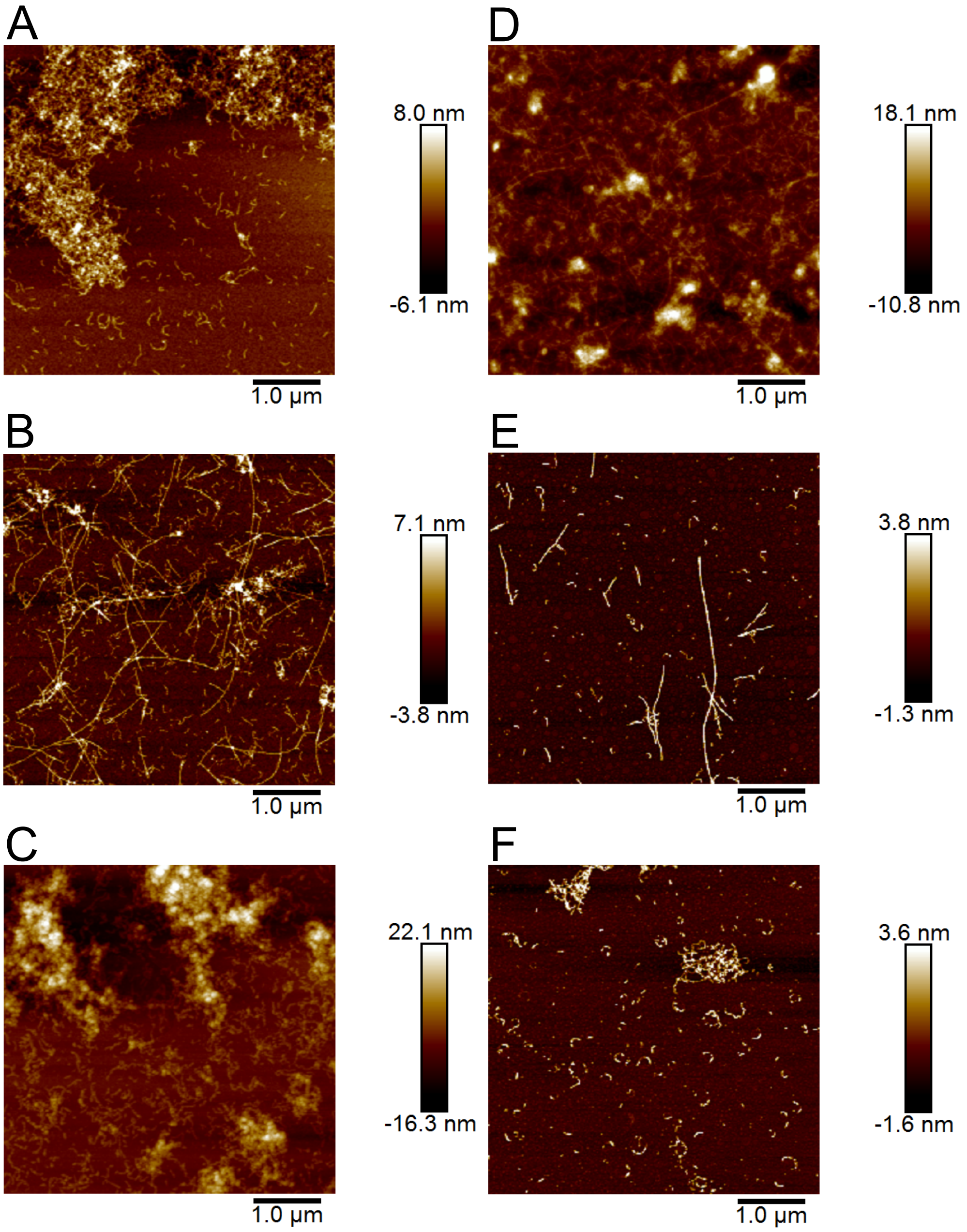

Figure S13. AFM images showing the cross-seeding ability of the two fibril morphologies. **(A-C)** Fibrillation of a sample with 77 g/l WPI. **(D-F)** Fibrillation of a sample with 40 g/l WPI. **(A,D)** No seeds added. **(B,E)** 5% of straight fibril seeds added. **(C,F)** 5% of curved fibril seeds added. Hence, cross-seeding has occurred in the samples shown in (B) and (F).

References

1. S. M. Loveday, X. L. Wang, M. A. Rao, S. G. Anema, L. K. Creamer and H. Singh, *Int Dairy J*, 2010, **20**, 571-579.
2. G. M. Kavanagh, A. H. Clark and S. B. Ross-Murphy, *Int J Biol Macromol*, 2000, **28**, 41-50.
3. A. M. Morris, M. A. Watzky, J. N. Agar and R. G. Finke, *Biochemistry*, 2008, **47**, 2413-2427.
4. A. Kroes-Nijboer, P. Venema, J. Bouman and E. van der Linden, *Langmuir*, 2011, **27**, 5753-5761.
5. G. Meisl, J. B. Kirkegaard, P. Arosio, T. C. Michaels, M. Vendruscolo, C. M. Dobson, S. Linse and T. P. Knowles, *Nat Protoc*, 2016, **11**, 252-272.
6. S. W. Cho, M. Gällstedt, E. Johansson and M. S. Hedenqvist, *Int J Biol Macromol*, 2011, **48**, 146-152.
